# Peer crowd-based targeting in E-cigarette advertisements: a qualitative study to inform counter-marketing

**DOI:** 10.1186/s12889-019-8126-x

**Published:** 2020-01-23

**Authors:** Minji Kim, Sarah Olson, Jeffrey W. Jordan, Pamela M. Ling

**Affiliations:** 1 Center for Tobacco Control Research and Education, University of California, San Francisco, 530 Parnassus Ave. Suite 366, San Francisco, CA 94143-1390 USA; 2 Rescue Agency, 2437 Morena Blvd., San Diego, CA 92110 USA

**Keywords:** E-cigarette advertisements, Peer crowd, Psychographic targeting, Targeted communication, Young adults

## Abstract

**Background:**

Cigarette lifestyle marketing with psychographic targeting has been well documented, but few studies address non-cigarette tobacco products. This study examined how young adults respond to e-cigarette advertisements featuring diverse peer crowds – peer groups with shared identities and lifestyles – to inform tobacco counter-marketing design.

**Methods:**

Fifty-nine young adult tobacco users in California participated in interviews and viewed four to five e-cigarette advertisements that featured characters from various peer crowd groups. For each participant, half of the advertisements they viewed showed characters from the same peer crowd as their own, and the other half of the advertisements featured characters from a different peer crowd. Advertisements were presented in random order. Questions probed what types of cues are noticed in the advertisements, and whether and how much participants liked or disliked the advertisements.

**Results:**

Results suggest that participants liked and provided richer descriptions of characters and social situations in the advertisements featuring their own peer crowd more than the advertisements featuring a different peer crowd. Mismatching age or device type was also noted: participants reported advertisements showing older adults were not intended for them. Participants who used larger vaporizers tended to dislike cigalike advertisements even if they featured a matching peer crowd.

**Conclusion:**

Peer crowd and lifestyle cues, age and device type are all salient features of e-cigarette advertising for young adults. Similarly, educational campaigns about e-cigarettes should employ peer crowd-based targeting to engage young adults, though messages should be carefully tested to ensure authentic and realistic portrayals.

## Background

Targeted communication aims to deliver appropriately designed messages to a pre-defined audience group that shares certain characteristics, making consolidated communication about the issue relevant to all members of the group. The messages are designed with specific characteristics in mind – including, but not limited to, demographics, cultural background, beliefs, behavioral tendencies, and risk factors. Therefore, targeted communication is more likely to achieve its persuasive goals than generic messaging as it makes messages more relevant and engaging for the target audience [1].

For effective message adaptation, it is important to define the target audience by segmenting the population into different subgroups with common needs and perspectives [2]. Compared to tailoring to the needs of each individual, targeted communication is less specific, but can be as effective if the target audience is well-defined without much variance in the targeted behavior [1]. However, a key question remains: What criteria should one use for effective audience segmentation?

Targeting by demographic factors, such as race/ethnicity, age groups, gender, or socioeconomic status, is one of the easiest and most salient strategies to adapt messages for a target audience. However, research such as that done by Boslaugh and colleagues has found that variables including self-efficacy, perceived barriers to engaging in the promoted behavior, and social support can be better predictors of health behavior than demographics [3]. Psychographic characteristics, such as attitudes, values, and lifestyles, can affect the audience’s social identity and perceived social norms by forming a reference or peer group [4], and thus can be effective variables for audience segmentation. When a person identifies with a certain peer group who shares certain characteristics, that individual may strive to reduce social uncertainty by learning about the social norms among the peer group, which in turn affects beliefs, attitudes and ultimately, behaviors via conformity to the group norm [4, 5]. Psychographic audience segmentation may help develop targeted messages that resonate with the given target audience by appealing to what they value the most, therefore yielding greater persuasive effects. Identification with peer groups defined based on psychographics was found to be a significant predictor of tobacco products among young adults [6, 7], suggesting its value as a meaningful segmentation criterion for tobacco-related communication [2].

### Peer crowd and its effect on tobacco use behavior

Adolescence and young adulthood are developmental periods when a young person strives to establish one’s identity [8], and during which time peer groups have a tremendous influence on the formation of self-concept and behavioral tendency [9]. ‘Peer crowds’ refer to groups with shared values, interests, lifestyles, styles of dress, influencers, and social tendencies and includes like-minded people outside of one’s immediate circle of friends [10–12]. Since peer crowds are connected to young adults’ social identities, lifestyle, and values, messages targeted to peer crowd may be more relevant than those adapted to demographic characteristics alone [7]. Peer crowd identification can affect young adults’ tobacco use as they accept the prototype and perceived social norms of their peer crowd to guide their own behavior [13]. Peer crowd identification has been shown to be significantly associated with young adults’ and adolescents’ risks and prevalence of tobacco use: those who identify with the ‘Hipster’ and ‘Hip Hop’ peer crowds have significantly greater likelihood of using tobacco [7, 11].

The tobacco industry has focused on psychographics when targeting young adults with different cigarette products and brands [14]. Recently, anti-tobacco campaigns are also starting to utilize psychographic targeting for certain subgroups associated with high risk of tobacco use. Targeted tobacco interventions and messages using peer crowds have been found to be an effective communication strategy [14–20]. Such strategy has been adopted in media campaigns including the FDA’s ‘Fresh Empire’ campaign targeting multi-cultural ‘Hip Hop’ urban youth [21], or the Virginia Foundation for Healthy Youth’s ‘Down and Dirty’ campaign targeting ‘Country’ teens [22] – although these programs have not yet been expanded to e-cigarettes.

### Importance of young adults in tobacco-related communication

Young adulthood is a critical time in tobacco-related communication – for both initiation and cessation of smoking cigarettes [23, 24]. Almost all tobacco users initiate before age 29 and smokers can avoid most of the adverse health consequences if they quit before age 30 [25, 26]. While cigarette smoking rates have substantially declined among young adults, the prevalence of e-cigarette use has rapidly increased [27], and current e-cigarette use among young adults (18–21 years: 16%; 22–24 years: 12%) was significantly higher than among older adults [28]. Youth and young adults are likely to be affected by their peer groups’ perception and prevalence of tobacco use, as well as targeted tobacco marketing [23]. Exposure to e-cigarette marketing may positively affect young adults’ perceptions of e-cigarettes [29, 30], subsequent use of e-cigarettes [31], as well as perceptions of regular cigarettes [32] and smoking urges [33, 34]. Understanding the effect of e-cigarette marketing is particularly important to inform tobacco control, as this understanding may facilitate identifying those who are most vulnerable to the effects of tobacco marketing, and why [6, 14].

The current study aimed to gain insight into two understudied issues related to peer crowd-based targeting: the effects on promotion rather than prevention of tobacco use, and use of peer crowd cues in e-cigarette advertisements, as most prior research has been limited to combustible cigarette marketing. In doing so, this study aimed to provide insights on what makes targeted e-cigarette marketing effective (or ineffective), and how counter-marketing messages may adopt effective psychographic targeting strategies.

The purpose of the study was to better understand how peer crowd matching impacts young adults’ responses to e-cigarette marketing, by specifically examining young adult responses to psychographic targeting present in e-cigarette marketing messages. We examined the salience of peer crowd and other cues (e.g., demographics, e-cigarette device type) when young adults gave qualitative descriptions as they viewed advertisements featuring matching and non-matching peer crowds. We also explored if and how peer crowd matching impacted liking of the advertisements, and how young adults accounted for similarities or differences between the peer crowds represented in the advertisements and themselves.

## Methods

Fifty-nine young adult tobacco users (18 to 29 years old) residing in California participated in an in-depth interview between January and August 2017. They were recruited through social media advertising. Eligible participants had used more than one tobacco product (cigarettes, e-cigarettes, and/or smokeless tobacco) within the past 30 days. Prior to the interview, participants completed a baseline survey that included the number of days they used each tobacco product during the past 30 days, socio-demographic characteristics, and peer crowd identification.

The semi-structured interviews lasted about 1 h and included both participants’ experience using multiple tobacco products and responses upon viewing a set of four to five e-cigarette print advertisements. Most interviews were conducted face-to-face, with a minority completed over the phone. Participants received a $100 gift card. This manuscript is based on the part of the interviews wherein the participants were presented with e-cigarette advertisements featuring various characters representing different peer crowd groups and discussed their responses to the advertisements. The procedures are described more in detail below.

### Baseline questionnaire

We measured age, gender, race/ethnicity, income, and education level. Peer crowd identification was measured using the I-Base Survey™ for which the scoring is described in detail elsewhere [7, 11, 15, 16, 18]. Briefly, the measure shows multiple pictures of young adults (36 males, 36 females) each pre-assigned to one of the six peer crowds: Mainstream, Young Professional, Hipster, Partier, Hip Hop, and Country (see Table 1 for the definition and example images corresponding to each peer crowd). Participants were asked to select three male and three female pictures that best fit their main group of friends and another three that least fit. The scores for each peer crowd were summed based on photo selection, and the highest peer crowd score was used to determine the participant’s peer crowd identification. If the participants’ responses resulted in a tie, their peer crowd was determined by randomly selecting one of the highest scored groups.

**Table 1:** Description of peer crowd groups and example images. Images courtesy of Rescue Agency, which has permission for use and grants permission for publication in this article

Peer crowd and description	Sample images representing crowd	Example advertisements
Mainstream: - Not motivated by social status - Prioritize family, career, or religion over socializing - Prefer a small number of close friends over many acquaintance		A. Blu magazine advertisement featuring a woman sitting indoors [35] B. Blu Instagram advertisement featuring two men and a woman sitting outdoors on dried leaves [36] C. VaporFi Instagram advertisement featuring a woman [37] D. Green Smoke website advertisement featuring a mature couple sitting together [38]
Young Professional: - Prioritize career and networking over partying - Won’t engage in behaviors that risk career - Dress well (not necessarily trendy)		E. Mistic website advertisement featuring four young adults in a meeting room [39] F. Ploom website advertisement featuring a White woman [40] G. NJOY Twitter advertisement featuring a man on a rooftop [41] H. Blow Vapor Facebook advertisement featuring a man wearing a brown suit in front of a tiled wall indoors [42]
Hip Hop: - Believe they have to overcome a disproportionate amount of struggles - Value strength, honor, and respect - Clothing/style are important status symbols		I. Blu magazine advertisement featuring a man in a casino [43] J. NJOY Instagram advertisement with two young adults outdoors [44] K. VaporFi Instagram advertisement with a female with dreadlocks in a car [45] L. VaporFi Instagram advertisement featuring a man sitting by a tree [46]
Hipster: - Counter-culture groups - Value individuality and creativity - Prioritize supporting local art, music and other creatives - Trendsetters		M. Blu magazine advertisement featuring a woman with tattooed arm [47] N. Blu Instagram advertisement featuring a young man with a nose ring [48] O. Fin magazine advertisement featuring a woman posing in front of an old-fashioned airplane [49] P. V2 Facebook advertisement featuring a man with sunglasses and moustache [50] Q. MarkTen magazine advertisement featuring an old man with sunglasses [51] R. ProVape Facebook advertisement with a senior woman wearing a large necklace [52]
Partier: - Highly value social status - Prioritize going out to bars and parties - Clothing and style are important status symbols, and take great care of their appearance		S. Blu magazine advertisement with three young men posing in a photo booth [53] T. Blu magazine advertisement with a young woman posing on a chair [54] U. Playboy magazine advertisement with a young man with many female hands and a close-up of a woman’s face [55] V. Cigavette Instagram advertisement with a young woman lying on a bed [56]

### Stimuli

The advertisements were all composed of both still images and text collected from print magazines and the internet. E-cigarette tobacco marketing databases including *Stanford Research into the Impact of Tobacco Advertisements* and *Trinkets &Trash* at Rutgers University were reviewed, as well as e-cigarette manufacturers’ websites and social media pages. All advertisements featured one or more human characters using the promoted products. Most characters were similar in age range to the young adult participants, but a few advertisements showing middle-aged or senior character(s) were added to the pool. The third and fourth authors have substantial prior experience with peer crowds in research [7, 11, 13, 15–18, 21, 22, 57], and reviewed each advertisement to identify peer crowds featured in each by consensus.

Participants saw 4–5 advertisements in a random order. [For the phone interview participants, a PDF containing e-cigarette advertisements in randomized order was sent via email prior to the interview. Participants were told not to open the file before the interview, and most of them viewed the advertisements on their phone while speaking with the interviewer. No participant reported difficulties viewing the images following this procedure.] Half of the advertisements featured characters from the peer crowd that the participants identify with (‘matching’), and the other half featuring a randomly selected different peer crowd (‘non-matching’). During the advertisement search process, no suitable e-cigarette advertisements featuring Country peer crowd characters were found. This may be because those who identify with the Country peer crowd are less likely to use e-cigarettes [7], and the Country image differs from the ‘techies’ or trendsetter images frequently used to promote e-cigarettes [58]. Therefore, seven participants who identified most strongly with Country peer crowd viewed their second highest scored peer crowd as the ‘matching’ peer crowd.

#### Changes in stimuli during the study

After about half of the interviews were completed, the authors decided to replace some advertisements, as participants were responding strongly to the type of the e-cigarette device depicted. E-cigarette devices have rapidly evolved. Early ‘first generation’ e-cigarettes resembled regular cigarettes (often called ‘cigalikes’), while ‘second/third generation’ devices referred to as ‘vape pens’, ‘tanks’, or ‘mods’, often feature larger batteries and more customizable parts to vary power or aerosol delivery [59]. The newer ‘pod vape’ devices frequently resemble a small USB stick (the most popular is JUUL), and are widely used by young adults [60]. Many young adults who used newer devices responded negatively toward advertisements for cigalikes. Most of the initial advertisements were for cigalikes, as the tobacco companies with larger marketing budgets [61] tended to manufacture cigalikes (e.g., Blu - Imperial, MarkTen - Altria; [62]). To ensure the participants could view a diverse array of devices, advertisements depicting newer generation devices were added, including one advertisement from JUUL.

### Interviews

Participants viewed the advertisements on an electronic tablet. After seeing an advertisement, participants were asked ‘what are the first few things you notice from the advertisement?’ The interviewer did not direct the participant’s attention to any specific aspect of the advertisement until they have finished discussing their first impression. This question was used to explore what features were the most salient to participants. Then, the interviewer asked more specifically about the characters shown in the advertisement. When necessary, additional probes were used to elicit responses to the characters and the advertisements, such as ‘do you think you would like (or be friends with) this person if you met them in real life?’ or to learn more about perceptions of the type of people in the advertisements, ‘what kind of job/car do you think these people have?’ Lastly, the interviewer asked ‘do you think this advertisement was made with people like you in mind? Why/Why not?’ Additional probes included asking what the participants would change to make the advertisement more relevant to them, what kind of people they think the advertisement was made for, or which advertisement was their ‘favorite’ and why. See the Additional file 1: for the interview guide.

During the conversation, the advertisement stayed on the screen, and participants could view or zoom in and out at will. When the discussion of an advertisement was finished, the participant moved on to the next advertisement by swiping the screen.

### Analyses

The first and second authors independently reviewed and coded all 59 transcripts using Dedoose™. First, two coders used a set of qualitative codes that were developed based on an initial reading of 10 transcripts. These two coders discussed and resolved any disagreements in face-to-face meetings to reach consensus, which was used in iterative modification of the codes. Transcripts were recoded as necessary following revisions to the codebook; all 59 transcripts were coded by two coders using this approach. The codes included: a) peer crowd cues (e.g. specific environments, contexts, and dress styles); b) demographic cues (age, race, and gender); c) the intended target audience; c) liking and disliking of the character; d) perceived ‘fakeness’ of the advertisements, including inauthentic portrayal of a peer group or obvious product promotion; and e) other information and features from the advertisements, including advertisement text, warning labels, and device types.

Individual differences emerged in terms of what cues were salient – for example, some participants mostly discussed the arguments and text, while others focused more on visual cues. To compare the differences in responses to advertisements featuring matching and non-matching peer crowds with the individual differences in mind, some analyses focused on the differences emerging between discussions of matching and non-matching peer crowd advertisements from the same participant, rather than between-participant comparison.

The two coders independently read the transcripts and assigned a quantitative score for liking of each advertisement for a participant in a procedure similar to Castro and colleagues’ ‘intensity scale coding’ [63], which refers to converting a code from a dichotomous mention of presence to an ordinal variable that reflects the intensity of emphasis and that can be further analyzed. Our scores ranged from 1 (strongly dislike the advertisement) to 6 (strongly like the advertisement). For example, if a participant strongly expressed liking the advertisement in general or the characters featured in the advertisements (e.g. ‘Dude looks really cool. Looks like a guy I want to grow up to be or hang out with’ – Aaron, 28 year old, male), or if the participant picked an advertisement as their favorite (e.g., ‘I actually like this advertisement, out of all of them’ – Blair, 28 year old, female), a higher score was assigned to the ad. On the other hand, discussion of dislike or cynicism (e.g. ‘Just a big old BS ad’ – Chris, 23 year old, male, Partier; ‘You can just tell he sucks’ – Danielle, 20 year old, female) resulted in lower scores. The two coders regularly met to discuss and resolve differences in the scale coding (typically if scores differed by more than 3 points). After that, the two rated scores for each advertisement were averaged, and the average of final scores of all matching vs. non-matching advertisements were compared across the participants.

## Results

Pseudonyms are used to protect participant confidentiality. The majority of the 59 participants were male (*n* = 45). About a third (*n* = 21) identified with Hipsters, followed by Partiers (*n* = 13) and Young professionals (*n* = 13). Table 2 shows the distribution of key demographic, peer crowd, and tobacco-related information.

**Table 2:** Descriptive statistics of the participants (*N* = 59)

Peer Crowd^a^	Total	Gender		Tobacco Products used in the past 30 days				
		Female	Male	Cig only^b^	E-cig only^b^	Cig & E-cig	Cig & SLT	Cig & E-cig & SLT
Hipster	23	9	14	3	1	12	0	7
Partier	16	1	15	1	0	6	2	7
Young Professional	13	2	11	0	0	7	1	5
Mainstream	5	1	4	0	0	4	0	1
Hip Hop	2	1	1	0	0	2	0	0
Total	59	14	45	4	1	31	3	20

*Cig* Cigarettes, *E-cig* E-cigarettes (including cigalikes, medium vapes/vape pens, or large vapes/tanks/mods), *SLT* Smokeless tobacco

^a^ Due to the unavailability of the e-cig advertisement targeting Country peer crowd, those who identified most strongly with Country were assigned to second-highest scored peer crowd

^b^ Participants who reported poly-use status at earlier screener, but single-use at baseline survey prior to the interview

### Participants responded more favorably to advertisements featuring the matching peer crowd

When seeing the advertisements featuring the ‘matching’ peer crowd, participants frequently acknowledged the similarities between themselves and the advertisement characters and found the characters and the advertisements more likable, relatable, and identifiable. Consequently, they tended to make favorable remarks about the advertisements and sometimes clearly asserting that they ‘liked’ them. In comparison to the non-matching peer crowds, participants described the messages as more attractive and convincing, and implicitly or explicitly acknowledged that the advertisements were intended for people like themselves.

This resulted in higher rated evaluation scores of matching (*M* = 3.76, *SD* = 1.51) than non-matching advertisements (*M* = 3.49, *SD* = 1.32), although the difference was not statistically significant (*t* = 1.60, *p* = .11). When asked to pick a ‘favorite’ advertisement among what they saw, 72.9% of participants selected a matching one. For example, seeing an advertisement featuring Partier men (Table 1-S), Ethan (18 year old, male, Partier) recognized and identified the ‘music festival’ Outside Land, in San Francisco, as the setting: ‘that’s relatable. That’s where I’m at right now [in life], and I like it’. Sometimes, a non-tobacco brand also shown in the advertisement worked as a cue of similar peer group membership and helped participants identify with the ad, as mentioned by Fiona (18 year old, female, Hip Hop) acknowledging an advertisement was meant for her as she noticed a guy wearing ‘Obey’ branded cap, which is ‘hype-y’ among her peers (Table 1-J).

As mentioned earlier, there were substantial individual differences in response. Many participants did not respond differently to advertisements featuring matching or non-matching peer crowd. While there were some people who always noticed peer crowd cues, others were more likely to mention demographics, or other information from the advertisements such as the copy text, product information, or warnings. However, a substantial subgroup of participants provided richer and more imaginative descriptions of the character’s lifestyle when viewing advertisements featuring the matching peer crowd. For example, Greg (19 year old, male, Partier) saw an advertisement featuring Partier female (Table 1-V) and described the character’s (likely) lifestyle in rich detail:

[T] his girl lives in an upscale LA neighborhood, drives like, probably a newer Audi, maybe a newer BMW … Definitely black, leather interior, like rims … She’s most likely a model and she’s probably pretty wealthy. And, she goes to a lot of celebrity parties … I grew up around these people in [Los Angeles].

On the other hand, seeing a Hipster advertisement, he focused more on the argument of the ad, saying ‘the caption says, enjoy your favorite product. So, he’s clearly enjoying his favorite product … the goal of this advertisement is to appeal to the cigarette market’. Similarly, Harry (18 year old, male, Partier) noticed peer crowd cues such as ‘party vibe’ and ‘coolness, sunglasses, summer, wind blowing her hair, elegance’ from advertisements featuring the matching (Partier) characters, but seeing a non-matching advertisement (Mainstream; Table 1-B) what he first mentioned was mostly demographic cues: ‘Three white people … hanging out, I see leaves fall. They are young’.

However, some participants seemed to resonate more with advertisements featuring non-matching peer crowds. Some Partier participants preferred seeing what they called ‘normal’ or ‘everyday’ people in the advertisements, because using e-cigarette was an everyday activity, rather than about partying. Kevin (21 year old, male, Hipster) saw himself maturing in the future including ‘looking for significant other’, and preferred an advertisement showing a Mainstream couple in bed (Table 1-D) more than the Hipster advertisements. In the following sections, we discuss additional message features that might interfere with liking advertisements with matching peer crowds.

### Authenticity is important in shaping the response toward advertisements

If participants felt that an advertisement was ‘staged’, or the characters were ‘pretentious’, or ‘posing’, it was considered unnatural and disliked even when the advertisement featured a matching peer crowd. On the other hand, participants liked advertisements that felt ‘candid’, ‘casual’, and capturing a real slice of life.

Similar to preferring candid images, participants often reacted negatively toward characters who did not *look like a ‘real smoker’* or like they were actually using the device. For example, Liam (24 year old, male, Young Professional) saw an advertisement with a Young Professional female with a vape pen (Table 1-F) and mentioned ‘she’s not really- she’s there just to hold it … She’s holding it kind of weird … there’s a little bit of, like, CG [computer graphic] vape … It clearly looks fake to me’. Matt’s (19 year old, male) first reaction to a cigalike advertisement was ‘She’s not a smoker … her teeth are too damn white to be smoking anything … also, you can tell, her fingers. Her fingers don’t look like she’s been biting at all’. If advertisements were perceived to be inauthentic participants were more likely to report that the advertisement was dishonest and manipulative.

Another aspect of authenticity was *plausibility of the behaviors* depicted in the advertisements. Seeing an advertisement featuring a couple wearing pajama-like white clothing in bed with the female holding an e-cigarette (Table 1-D), Noah’s (29 year old, male, Mainstream) first remark was that ‘no one does that from my experience. No one goes, like - just smoking. I don’t know, I don’t smoke in my bed, that’s just weird.’ Seeing another advertisement depicting young business people using e-cigarettes inside a meeting room (Table 1-E), two Young professional participants, Liam (24 year old, male) and Olivia (23 year old, female) remarked that this is not something one would or allowed to do in reality, thus undermining the effectiveness of the ad.

However, not every participant perceived the same images as inauthentic; when viewing the Young Professional advertisement mentioned above (Table 1-E), Pearl (21 year old, female, Young professional)‘s first mention was ‘I’m seeing a trendy, young, start-up company with all the people at a business meeting all smoking e-cigarettes that also look like real cigarettes as well. So, I think of the new age version of “Mad Men” when I see this’.

Authentic portrayal of the peer crowd was also crucial in engaging the intended target audience. Advertisements that were not successful in this aspect were seen as ‘clichéd’ and ‘trying too hard’, a point which was raised largely by the Hipster participants. For example, an advertisement featuring a male using a box mod with a moustache, aviator sunglasses, tight t-shirt and jacket (Table 1-P) was criticized by Hipsters for using a shallow display of Hipster clichés including careful hair grooming, outfits, and large accessories like sunglasses and watches. Aaron (28 year old, male, Hipster) said ‘it’s supposed to be serious but it’s like it’s so cliché ... That’s like so quintessential Hipster f***ing dude... He’s trying real hard to be cool. He’s not himself’. Another Hipster, Quinton (21 year old, male), said of the same ad, ‘[He looks like] full of himself … He’s trying to look really cool … maybe it was [made with people like me in mind], but they were wrong … That’s not my kind of guy’.

There were also different opinions even within the same peer crowd audience about what is considered a cliché vs. authentic. Seeing the Hipster advertisement hated by some Hipsters (Table 1-P), Robert (23 year old, male, Hipster) mentioned that ‘definitely targeted to the millennial culture, like what you would call the hipster … given his haircut, his sunglasses, his beard, the blazer, white T-shirt, all of that, the styling, everything about it’. Another Hipster Shannon (26 year old, female) acknowledged that the advertisement was ‘definitely’ made with someone like herself in mind because ‘the accessorizing, the, again, the beard, the facial hair. That’s something that hipsters are always talking about’.

### Device type featured in the advertisements is important

The type of the device promoted in the advertisement also appeared to influence how participants reacted to the advertisements, especially cigalikes. Participants were quick to recognize device types. When asked about the first few things that stood out in the ad, most participants started by describing the human character or the setting; but many also turned their attention to the device. Those who were using larger vaporizers seemed committed to this device – more than half of the large vaporizer users who made any comments on device type expressed negative perceptions of cigalikes, and subsequently disliked advertisements promoting cigalikes regardless of which peer crowd was featured. Chris (23 year old, male, Partier; using cigarette/large vaporizer/smokeless tobacco) saw a cigalike advertisement with a female Partier (Table 1-T) and mentioned:

I don’t really see the appeal in this either. I just think it’s because my opinion is a little bit biased because I hate these little things [indicating the device] … I think they’re like the biggest waste, I think they’re stupid. They didn’t really work.

Such accounts indicate that personal experiences with products informed participants’ perception of manufacturers and subsequently, to the advertisements. For example, as a daily user of large vaporizers, Tim found ‘tanks’ and ‘mods’ were ‘more effective than the cigarette looking ones’. Ian (using cigarette/large vaporizer/smokeless tobacco) remembered seeing Blu cigalike marketing in TV and seeing their products in ‘pretty much every store’, but mentioned that cigalikes are ‘garbage’, of ‘very low quality’ and left a bad taste ‘like burnt popcorn’, which drove him to use larger vaporizers.

The visual resemblance sometimes caused some cigalike advertisements to be mistaken for cigarette advertisements, and this seemed to bring back negative perceptions and social stigmas associated with smoking cigarettes, such as the behavior of throwing away the ‘butts’ or the disposable device after use, or the history of manipulative marketing by ‘big tobacco’. This seems to be especially true for large vaporizer users: almost all negative remarks about cigalikes were made by large vaporizer users, except for one medium vape pen user. On the other hand, other device users may have confused the device as combustible cigarette but did not necessarily describe it in a negative light. Some participants used the term ‘e-cigarettes’ exclusively for cigalikes, while calling others ‘vapes’ or ‘vaporizers’.

Seeing a cigalike advertisement with a Hipster female character (Table 1-M), Tessa (using cigarette/medium and larger vaporizer) remarked that ‘She’s smoking that Blu, nasty e-cig’, explaining that she did not like cigalikes because they were ‘really wasteful’ as most of them were disposable rather than rechargeable and ‘literally the same thing as smoking cigarettes’. Uriel (using cigarette/large vaporizer), felt a cigalike advertisement was very ‘corporate’ and ‘money making’, while the ‘vape specific companies’ have ‘a community behind’ them, so ‘they don’t really try to advertise it’ but rather relying on customer-generated reviews. Participant accounts suggest that cigalikes were also considered to be a product for those who were less experienced with e-cigarettes:

[P] eople who smoke Blus, I feel like they don’t know what they’re doing. Like, they don’t know, they haven’t done the research... oh look, there’s an ad, I saw that, let me go try these out. … I feel like they just got sucked in. (Noah, cigarette/large vaporizer user)

### Demographics cues may also affect identification with matching peer crowd advertisements

The first things that participants mentioned they noticed in the advertisements included both peer crowd cues, such as dress styles, environments, or lifestyles (202 times) and demographic cues such as race, age, and gender (178 times), and many responses included both demographic and peer crowd cues (134 times). However, the discussions of peer crowd cues tend to be richer than discussion of demographics which were often limited to short adjectives like ‘male/female’, ‘guys’, ‘ladies’, ‘old/young’, or ‘White/Black’ - which might be interpreted as peer crowds having greater salience to respondents. For example, Kevin (21 year old, male, Hipster) described an advertisement featuring a dressed-up male (Table 1-P) with more emphasis on peer crowd cues than demographics:

There’s like, a business model type. Has, you know, wise elegancy, definitely clean cut [peer crowd cues]. So, I feel like this would target me more as the other one [featuring a Mainstream female at home], based on the representation of the male [demographic cue], clean cut, business type model [peer crowd cues].

For some people, a matching peer crowd with mismatched demographic group still generated favorable responses. For example, when viewing another advertisement with a Hipster female (Table 1-M), a male Hipster participant remarked:

[E] ven though the girl's a little bit older, she still looks pretty relatable to people like me. … Tattoos make me think she's, like - she doesn't really - like, even if people don't like tattoos, it's very visible, so it doesn't really matter to her. – Victor (25 year old, male, Hipster)

However, many participants were also quite attuned to the age of the advertisement characters. Advertisements featuring older adults were more difficult to relate to. William (20 year old, male, Partier) described an advertisement with a senior man using e-cigarette (Table 1-Q) as:

It's an older gentleman of sorts. It seems like he would be a cool grandpa. … I would say [this is for] more older adults. Adults maybe, like, 30s, 40s, 50s, 60s, and so on. The photo also looks like he's around those ages as well.

Even when seeing an advertisement featuring a matching peer crowd, which might garner a more favorable response, age difference seemed to interfere with identification with the character and the message. Seeing an advertisement with a senior Hipster female (Table 1-R), Xena (24 year old, female, Hipster) mentioned ‘I am noticing that is an older woman, which is unusual for a vaping advertisement … definitely not something targeted towards me, obviously’.

### Race/ethnicity-related cues are not as salient as age

Unlike other psychographic or demographic cues, participants did not regularly remark on the race/ethnicity of characters in the advertisements. Unlike age, it was not clear whether mismatch in race and ethnicity between the advertisement character and participants negatively affected reactions to matching peer crowd advertisements. When mentioned, it often involved responding to advertisements featuring non-white characters, mentioning that it is uncommon to see a person of color using e-cigarettes or featured in e-cigarette advertisements (e.g., ‘I have never seen a Black guy smoking a JUUL’, Yoel, Hispanic). Noticing a non-White character in the advertisements was sometimes connected to the perception that the advertiser is showing a diverse group to appeal to a wider audience – which was perceived both positively and negatively. Some people thought the advertisement was inclusive, but some reacted negatively to the intentional marketing. Zachary (Hispanic) criticized an advertisement showing both male and female, and White and Black characters (Table 1-E): ‘Definitely, they’ve strategically placed the Black dude, very front and center, like “This is for everyone” … This guy, he just looks like a tool’. Participants of minority race/ethnicity were slightly more likely to mention race/ethnicity when discussing what they first noticed from the advertisements: 65% of our sample were either non-White or Hispanic, and they made 16 of the 21 first mention excerpts (76%) that recognized the featured characters’ race/ethnicity.

## Discussion

In-depth interviews with young adults discussing their responses to e-cigarette advertisements revealed that advertisements with a matching peer crowd had more favorable responses and more elaborate descriptions, as long as the representation of characters and contexts was perceived as authentic. This suggests that peer crowd-related cues are salient and noticeable for young adult audiences. In addition, device type also played an important role; some participants expressed strong unfavorable attitudes toward cigalikes, which affected their advertisement evaluation, regardless of peer crowd matching. The negative perceptions of cigarettes and the tobacco industry seemed to cast a shadow on the cigalikes. Visual resemblance to cigarettes and being disposable (rather than rechargeable) elicited negative responses towards cigalike advertisements. Some of the negative reactions may have been informed by the fact that big tobacco companies manufactured mainly cigalikes at the time of the study [62], while ‘independent’ vape shops were the source for large vaporizers [64].

Demographic cues were mentioned slightly less frequently than peer crowd related cues, but emerged as another salient factor – especially age. Older characters were often interpreted as signals that the advertisement was not intended for the young adult participants. On the other hand, race/ethnicity appear to be less salient than other demographics or psychographic cues in this study. Although race/ethnicity was mentioned infrequently, many expressed surprise to see non-White characters featured in e-cigarette advertisements, which is in line with previous findings on African American adolescents’ perceptions of e-cigarettes [58], or the fact that among US adults, non-Hispanic White adults are more likely to use e-cigarette than Hispanics or non-Hispanic Blacks [65]. Participants of minority race/ethnicity were slightly more likely to mention race/ethnicity when discussing their first reactions to the advertisements, which is consistent with studies showing for minority college students viewing matching race was valued more than White students (e.g., [66]).

### Implications for tobacco control

These young adult responses to e-cigarette advertisements might inform tobacco control and counter-marketing message design. Peer crowd-based targeting and other contextual cues may be useful strategies to increase the salience of anti-tobacco messages for young adults. In research lab settings, peer crowd-based targeting strategies have shown significant effects on attitudes and intentions related to smoking [19, 20]. Beyond the previously mentioned ‘Fresh Empire’ and ‘Down and Dirty’ campaigns that target Hip Hop and Country youth respectively, interventions targeting Hipsters [18] and Partiers [15] in bars and clubs have shown significant decreases in cigarette smoking, but these interventions have not addressed e-cigarette use. This study suggests that peer crowd-based targeting may be useful for e-cigarette counter-marketing messages.

Our findings suggest authenticity is a crucial factor in targeting a certain peer crowd; otherwise, targeted advertisements may be rejected, or even ridiculed. Participants in this study noted how implausible situations that were unlikely to be encountered in real life, or images that appeared to be ‘staged’ evoked perceptions of manipulation to sell products. Clichés, such as images seen as stereotypes of certain groups (e.g., Hipsters – unique hairstyles; Young Professionals – boardroom meeting), were perceived as a failed attempt of targeted marketing by many. Particularly since the Hipster peer crowd most often values individuality and authenticity [18], participants identifying with this peer crowd reacted most negatively to stereotypes or commercial appropriation of their group.

The discussion of clichés suggests that using visual features alone to represent an intended target peer crowd may not be sufficient; other message features, such as the argument, should reflect values of the intended target group. Recruiting community members of the target audience to generate creative content can be one way to achieve authenticity. For example, the ‘COMMUNE’ intervention commissioned young adult Hipster artists to create anti-tobacco messaging; the resultant art frequently included social justice themes relevant to Hipsters [18]. This widely used tactic in community-based participatory research (e.g., [67, 68]) could be leveraged for e-cigarette counter-marketing campaigns and interventions. Authenticity can also be improved through rigorous pre-testing with the target audience [69] to detect psychological reactance and negative emotions.

This study also suggests that e-cigarette educational campaigns should be careful to portray the correct type of e-cigarette device for the target audience, especially if messages are for current users. Many e-cigarette users have cultural associations and strong opinions about different device types and their users. Many experienced e-cigarette users progress to larger vaporizers over time [70] and perceived cigalikes as a product for novices [71]; on the other hand, mods and tanks are considered for ‘techies’ and ‘hobbyists’ who were more experienced and knowledgeable, drawn to the technical aspects of the device, and customization options [72–74]. Among our participants, more than half of the large vaporizer users who made any comments about the types of devices made negative comments about cigalike devices and disliked the advertisements even if the advertisement featured matching peer crowd characters. Consumers often seek self-authentication by consuming brands that are believed to be connected to culture and community [75], preferences for large vaporizers were consistent with the belief that these products are produced by enthusiastic small businesses (not big tobacco companies). Therefore, portraying the right kind of the device is another aspect of authenticity. As many youth and young adults are using pod-type and large vaporizers as shown in relative decrease in market share by well-known cigalike brands (e.g., Blu, Vuse, MarkTen) when compared to JUUL since 2017 [60], using cigalikes in an anti-e-cigarette message would be less engaging or relevant. Moreover, portraying a tech-savvy or trend-setting young adult using cigalike device might raise suspicions among young adult audiences as it does not match what they would use in real life.

The current study also suggests that featuring young models in e-cigarette advertisements may be more appealing to young adults. The FDA has announced plans to regulate youth-targeted sales and marketing of e-cigarettes, including JUUL [76]. In response to the criticism, JUUL started a new ‘#SwitchToJUUL’ campaign featuring testimonials from older adult former-smokers in May 2018 and shut down their social media accounts in November 2018 – although much unofficial content on the internet featuring adolescent and young adult users remains. In addition to peer crowd and contextual cues, age was frequently mentioned and discussed when young adults in this study responded to e-cigarette advertisements. Seeing older adults was an apparent cue that the advertisements were not intended for young adults, and in many cases, young adults expressed less interest in and less favorable responses toward such messages. JUUL recently changed their advertising campaign to feature older adults; this study suggests this change might reduce appeal to young adults. However, the brand has already been established using youthful advertising and it is already widely perceived as a youthful product; this perception may not be affected by subsequently featuring older adults in advertising. Whether a change in advertising that is not accompanied by other actions to reduce youth access to the product actually results in reduced use by youth and young adults is an empirical question that deserves further rigorous examination.

### Limitations

Participants were all young adult poly-tobacco users residing in California, and thus findings cannot be extended to a larger population. While the results suggest important implications for youth-targeted marketing, the current study recruited young adults, not adolescents – thus limiting the generalizability. Also, all participants were current users of multiple tobacco products, who may have been savvier than single product users or non-users, and more likely to recognize the devices depicted in advertisements.

All advertisements used in the study were from magazines or online (website or social media). Another active venue for the tobacco industry is point-of-sale marketing. Exposure to point-of-sale tobacco marketing was shown to be a predictor of increased tobacco use and purchase behaviors, as well as decreased quitting attempts [77–81]. Future studies examining targeting strategies in point-of-sale e-cigarette marketing and their effects on youth and young adults would provide important insights relevant to regulating tobacco marketing.

Studies using national samples or experimental designs to evaluate the effects of tobacco-related communication using peer crowd-based targeting would complement this study to inform improved counter-marketing message design and regulation of tobacco marketing. Also, future studies could examine how e-cigarette advertisements targeting different peer crowds interact with tobacco use status, as the effects of targeted marketing may differ for those not using tobacco products.

## Conclusion

This study provides important insights into how young adult poly-tobacco users responded to peer crowd-based targeting in e-cigarette advertisements, and this can be transferred to designing effective counter-marketing campaigns. Peer crowd-related cues were highly salient and matching peer crowd and age resulted in more favorable responses. However, for successful peer crowd-based targeting, messages must be perceived as authentic, particularly with the portrayal of realistic situations and device types for the target audience.

## Supplementary information

**Additional file 1.** Interview guide. Includes instructions for the interview and questions asked to the participants during the interview.

## Data Availability

The dataset is qualitative and includes numerous quotes from which participants are potentially identifiable. For this reason, the raw dataset will not be available.

## Abbreviations

Cig.: Cigarettes
E-cig: E-cigarettes (including cigalikes, medium vapes/vape pens, or large vapes/tanks/mods)
SLT: Smokeless tobacco
