# Supplementary material for: Peer crowd-based targeting in E-cigarette advertisements: a qualitative study to inform counter-marketing
Source: BMC Public Health. 2020 Jan 23;20:32. doi: 10.1186/s12889-019-8126-x (PMC6977342; doi:10.1186/s12889-019-8126-x)
Supplement: Supplementary file 1 — Additional file 1. Interview guide. Includes instructions for the interview and questions asked to the participants during the interview. [file 12889_2019_8126_MOESM1_ESM.docx]

**Peer Crowd-Based Targeting in E-Cigarette Advertisements:
A qualitative study to inform counter-marketing**

**Supplement file: Interview guide for the ad viewing**

**Instruction to the interviewer:

In-person interview: Appropriate ads will be queued up by second staff person on iPad.

Phone interview: Appropriate ads will be sent to the participant via email by staff.

**For each ad:**

- **What are the first things you notice from this ad?**
- **What do you think of the characters shown in the ad?**
- **Do you think this ad was made with people like you in mind? Why?**
  - Guide people to point out specific things that they “identify” with the character in the ad.
  - **(If No) Who do you think this ad is for?**
- Additional prompts if participant is having difficulty articulating answers:
  - Do you think you will like this person if you meet him/her/them in your real life?
  - What kind of job do you think he/she/they have?
  - What kind of car do you think he/she/they drive?
  - (If they seem to NOT like the ads) What would you change about this ad so that this appeals to you more?

**After all ads have been shown:**

- Among the ads we have seen, what is your favorite ad (or, which ad appeals to you the most)?
- (optional) If we think only about the people we have seen, who did you like the best among those you’ve seen here?
